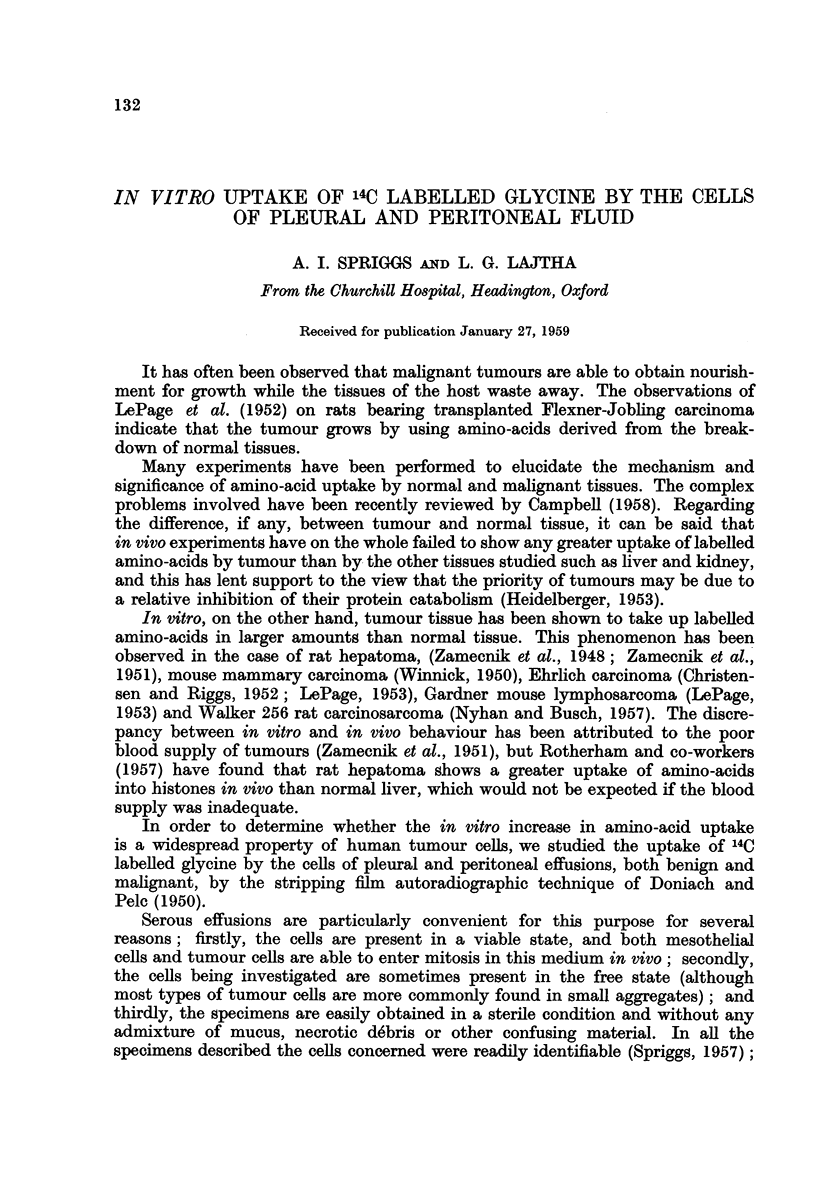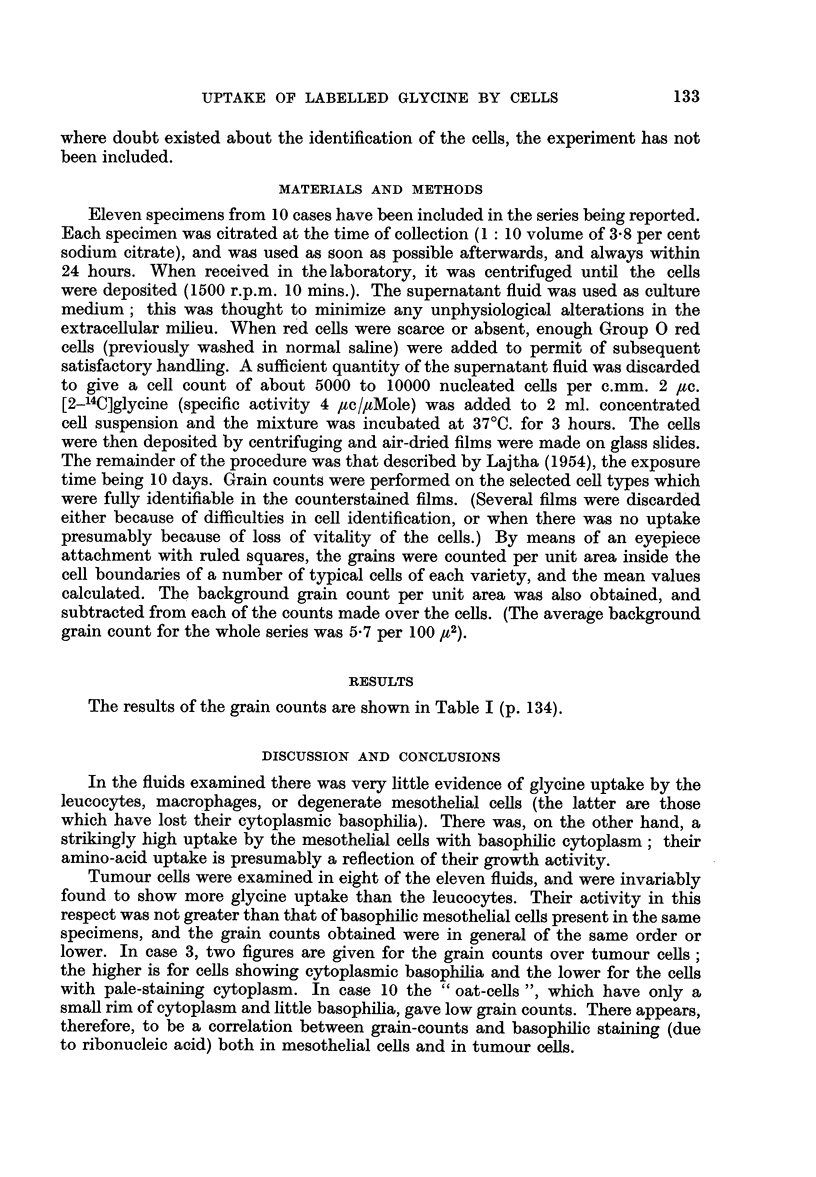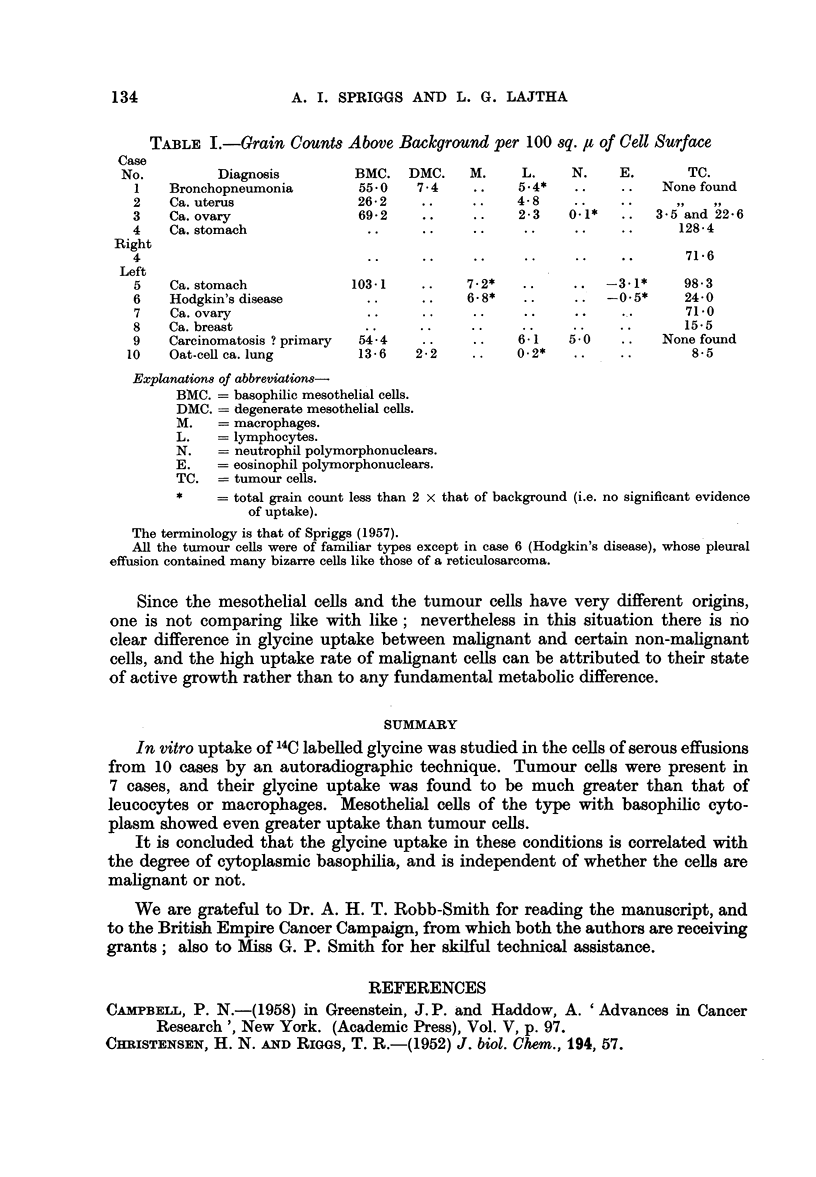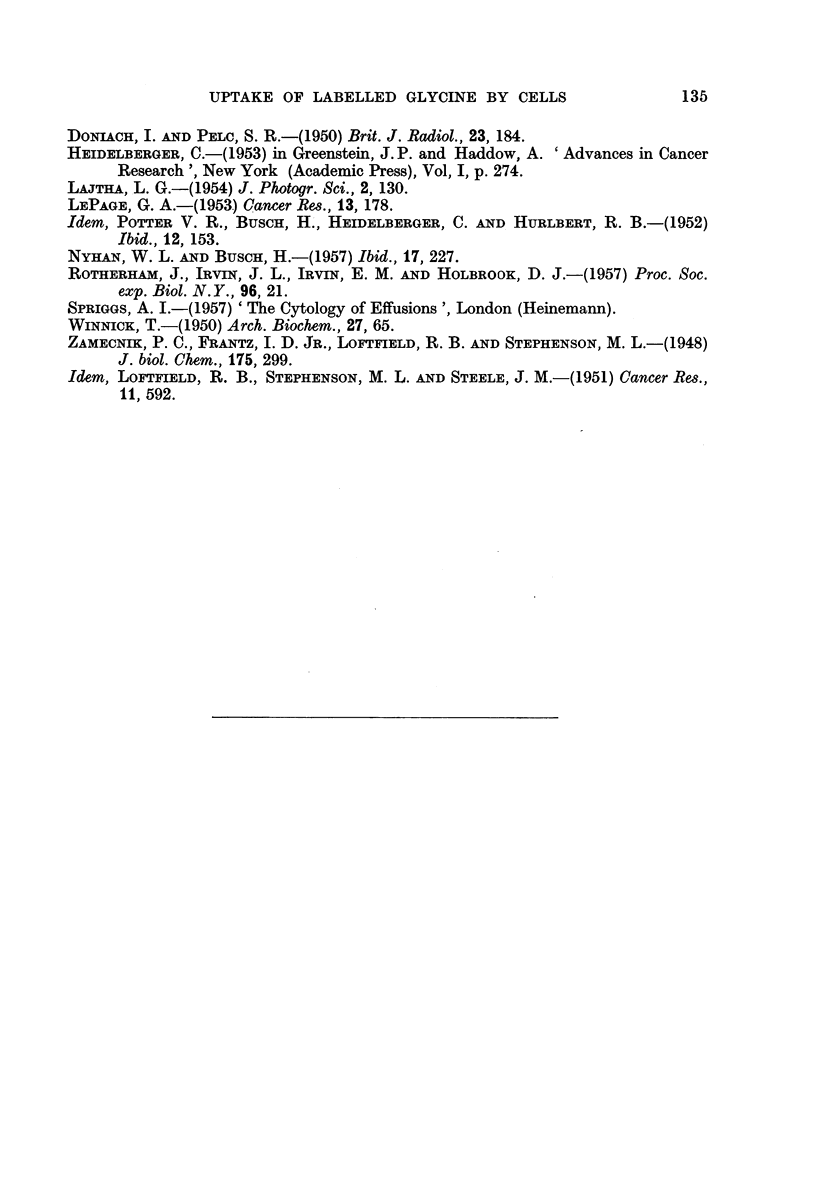# In vitro Uptake of ^14^C Labelled Glycine by the Cells of Pleural and Peritoneal Fluid

**DOI:** 10.1038/bjc.1959.19

**Published:** 1959-03

**Authors:** A. I. Spriggs, L. G. Lajtha


### *IN VITRO* UPTAKE OF $^{14}C$ LABELLED GLYCINE BY THE CELLS OF PLEURAL AND PERITONEAL FLUID

A. I. SPRIGGS AND L. G. LAJTHA

*From the Churchill Hospital, Headington, Oxford*

Received for publication January 27, 1959

It has often been observed that malignant tumours are able to obtain nourishment for growth while the tissues of the host waste away. The observations of LePage *et al.* (1952) on rats bearing transplanted Flexner-Jobling carcinoma indicate that the tumour grows by using amino-acids derived from the breakdown of normal tissues.

Many experiments have been performed to elucidate the mechanism and significance of amino-acid uptake by normal and malignant tissues. The complex problems involved have been recently reviewed by Campbell (1958). Regarding the difference, if any, between tumour and normal tissue, it can be said that *in vivo* experiments have on the whole failed to show any greater uptake of labelled amino-acids by tumour than by the other tissues studied such as liver and kidney, and this has lent support to the view that the priority of tumours may be due to a relative inhibition of their protein catabolism (Heidelberger, 1953).

*In vitro*, on the other hand, tumour tissue has been shown to take up labelled amino-acids in larger amounts than normal tissue. This phenomenon has been observed in the case of rat hepatoma, (Zamecnik *et al.*, 1948 ; Zamecnik *et al.*, 1951), mouse mammary carcinoma (Winnick, 1950), Ehrlich carcinoma (Christensen and Riggs, 1952 ; LePage, 1953), Gardner mouse lymphosarcoma (LePage, 1953) and Walker 256 rat carcinosarcoma (Nyhan and Busch, 1957). The discrepancy between *in vitro* and *in vivo* behaviour has been attributed to the poor blood supply of tumours (Zamecnik *et al.*, 1951), but Rotherham and co-workers (1957) have found that rat hepatoma shows a greater uptake of amino-acids into histones *in vivo* than normal liver, which would not be expected if the blood supply was inadequate.

In order to determine whether the *in vitro* increase in amino-acid uptake is a widespread property of human tumour cells, we studied the uptake of $^{14}C$ labelled glycine by the cells of pleural and peritoneal effusions, both benign and malignant, by the stripping film autoradiographic technique of Doniach and Pelc (1950).

Serous effusions are particularly convenient for this purpose for several reasons ; firstly, the cells are present in a viable state, and both mesothelial cells and tumour cells are able to enter mitosis in this medium *in vivo* ; secondly, the cells being investigated are sometimes present in the free state (although most types of tumour cells are more commonly found in small aggregates) ; and thirdly, the specimens are easily obtained in a sterile condition and without any admixture of mucus, necrotic débris or other confusing material. In all the specimens described the cells concerned were readily identifiable (Spriggs, 1957) ; where doubt existed about the identification of the cells, the experiment has not been included.

### MATERIALS AND METHODS

Eleven specimens from 10 cases have been included in the series being reported. Each specimen was citrated at the time of collection (1 : 10 volume of 3·8 per cent sodium citrate), and was used as soon as possible afterwards, and always within 24 hours. When received in the laboratory, it was centrifuged until the cells were deposited (1500 r.p.m. 10 mins.). The supernatant fluid was used as culture medium ; this was thought to minimize any unphysiological alterations in the extracellular milieu. When red cells were scarce or absent, enough Group O red cells (previously washed in normal saline) were added to permit of subsequent satisfactory handling. A sufficient quantity of the supernatant fluid was discarded to give a cell count of about 5000 to 10000 nucleated cells per c.mm. 2 $\mu$c. [2–$^{14}$C]glycine (specific activity 4 $\mu$c/$\mu$Mole) was added to 2 ml. concentrated cell suspension and the mixture was incubated at 37°C. for 3 hours. The cells were then deposited by centrifuging and air-dried films were made on glass slides. The remainder of the procedure was that described by Lajtha (1954), the exposure time being 10 days. Grain counts were performed on the selected cell types which were fully identifiable in the counterstained films. (Several films were discarded either because of difficulties in cell identification, or when there was no uptake presumably because of loss of vitality of the cells.) By means of an eyepiece attachment with ruled squares, the grains were counted per unit area inside the cell boundaries of a number of typical cells of each variety, and the mean values calculated. The background grain count per unit area was also obtained, and subtracted from each of the counts made over the cells. (The average background grain count for the whole series was 5·7 per 100 $\mu^2$).

### RESULTS

The results of the grain counts are shown in Table I (p. 134).

### DISCUSSION AND CONCLUSIONS

In the fluids examined there was very little evidence of glycine uptake by the leucocytes, macrophages, or degenerate mesothelial cells (the latter are those which have lost their cytoplasmic basophilia). There was, on the other hand, a strikingly high uptake by the mesothelial cells with basophilic cytoplasm ; their amino-acid uptake is presumably a reflection of their growth activity.

Tumour cells were examined in eight of the eleven fluids, and were invariably found to show more glycine uptake than the leucocytes. Their activity in this respect was not greater than that of basophilic mesothelial cells present in the same specimens, and the grain counts obtained were in general of the same order or lower. In case 3, two figures are given for the grain counts over tumour cells ; the higher is for cells showing cytoplasmic basophilia and the lower for the cells with pale-staining cytoplasm. In case 10 the " oat-cells ", which have only a small rim of cytoplasm and little basophilia, gave low grain counts. There appears, therefore, to be a correlation between grain-counts and basophilic staining (due to ribonucleic acid) both in mesothelial cells and in tumour cells.

TABLE I.—*Grain Counts Above Background per* 100 *sq.* $\mu$ *of Cell Surface*

| Case No. | Diagnosis | BMC. | DMC. | M. | L. | N. | E. | TC. |
|---|---|---|---|---|---|---|---|---|
| 1 | Bronchopneumonia | 55·0 | 7·4 | .. | 5·4* | .. | .. | None found |
| 2 | Ca. uterus | 26·2 | .. | .. | 4·8 | .. | .. | " " |
| 3 | Ca. ovary | 69·2 | .. | .. | 2·3 | 0·1* | .. | 3·5 and 22·6 |
| 4 Right | Ca. stomach | .. | .. | .. | .. | .. | .. | 128·4 |
| 4 Left | | .. | .. | .. | .. | .. | .. | 71·6 |
| 5 | Ca. stomach | 103·1 | .. | 7·2* | .. | .. | −3·1* | 98·3 |
| 6 | Hodgkin's disease | .. | .. | 6·8* | .. | .. | −0·5* | 24·0 |
| 7 | Ca. ovary | .. | .. | .. | .. | .. | .. | 71·0 |
| 8 | Ca. breast | .. | .. | .. | .. | .. | .. | 15·5 |
| 9 | Carcinomatosis ? primary | 54·4 | .. | .. | 6·1 | 5·0 | .. | None found |
| 10 | Oat-cell ca. lung | 13·6 | 2·2 | .. | 0·2* | .. | .. | 8·5 |

*Explanations of abbreviations*—
BMC. = basophilic mesothelial cells.
DMC. = degenerate mesothelial cells.
M. = macrophages.
L. = lymphocytes.
N. = neutrophil polymorphonuclears.
E. = eosinophil polymorphonuclears.
TC. = tumour cells.
* = total grain count less than 2 × that of background (i.e. no significant evidence of uptake).

The terminology is that of Spriggs (1957).
All the tumour cells were of familiar types except in case 6 (Hodgkin's disease), whose pleural effusion contained many bizarre cells like those of a reticulosarcoma.

Since the mesothelial cells and the tumour cells have very different origins, one is not comparing like with like ; nevertheless in this situation there is no clear difference in glycine uptake between malignant and certain non-malignant cells, and the high uptake rate of malignant cells can be attributed to their state of active growth rather than to any fundamental metabolic difference.

### SUMMARY

*In vitro* uptake of $^{14}C$ labelled glycine was studied in the cells of serous effusions from 10 cases by an autoradiographic technique. Tumour cells were present in 7 cases, and their glycine uptake was found to be much greater than that of leucocytes or macrophages. Mesothelial cells of the type with basophilic cytoplasm showed even greater uptake than tumour cells.

It is concluded that the glycine uptake in these conditions is correlated with the degree of cytoplasmic basophilia, and is independent of whether the cells are malignant or not.

We are grateful to Dr. A. H. T. Robb-Smith for reading the manuscript, and to the British Empire Cancer Campaign, from which both the authors are receiving grants ; also to Miss G. P. Smith for her skilful technical assistance.

### REFERENCES

CAMPBELL, P. N.—(1958) in Greenstein, J.P. and Haddow, A. ' Advances in Cancer Research ', New York. (Academic Press), Vol. V, p. 97.
CHRISTENSEN, H. N. AND RIGGS, T. R.—(1952) *J. biol. Chem.*, **194**, 57.

Doniach, I. and Pelc, S. R.—(1950) *Brit. J. Radiol.*, **23**, 184.
Heidelberger, C.—(1953) in Greenstein, J.P. and Haddow, A. ‘ Advances in Cancer Research ’, New York (Academic Press), Vol, I, p. 274.
Lajtha, L. G.—(1954) *J. Photogr. Sci.*, **2**, 130.
LePage, G. A.—(1953) *Cancer Res.*, **13**, 178.
*Idem*, Potter V. R., Busch, H., Heidelberger, C. and Hurlbert, R. B.—(1952) *Ibid.*, **12**, 153.
Nyhan, W. L. and Busch, H.—(1957) *Ibid.*, **17**, 227.
Rotherham, J., Irvin, J. L., Irvin, E. M. and Holbrook, D. J.—(1957) *Proc. Soc. exp. Biol. N.Y.*, **96**, 21.
Spriggs, A. I.—(1957) ‘ The Cytology of Effusions ’, London (Heinemann).
Winnick, T.—(1950) *Arch. Biochem.*, **27**, 65.
Zamecnik, P. C., Frantz, I. D. Jr., Loftfield, R. B. and Stephenson, M. L.—(1948) *J. biol. Chem.*, **175**, 299.
*Idem*, Loftfield, R. B., Stephenson, M. L. and Steele, J. M.—(1951) *Cancer Res.*, **11**, 592.